# Factors associated with satisfaction and perceived helpfulness of mental healthcare: a World Mental Health Surveys report

**DOI:** 10.1186/s13033-024-00629-7

**Published:** 2024-03-01

**Authors:** Meredith G. Harris, Alan E. Kazdin, Richard J. Munthali, Daniel V. Vigo, Dan J. Stein, Maria Carmen Viana, Sergio Aguilar-Gaxiola, Ali Al-Hamzawi, Jordi Alonso, Laura Helena Andrade, Brendan Bunting, Stephanie Chardoul, Oye Gureje, Chiyi Hu, Irving Hwang, Elie G. Karam, Fernando Navarro-Mateu, Daisuke Nishi, Ricardo Orozco, Nancy A. Sampson, Kate M. Scott, Cristian Vladescu, Bogdan Wojtyniak, Miguel Xavier, Zahari Zarkov, Ronald C. Kessler

**Affiliations:** 1 School of Public Health, The University of Queensland, c/o QCMHR, Locked Bag 500, Archerfield, QLD 4108 Australia; 2 Queensland Centre for Mental Health Research, The Park Centre for Mental Health, Wolston Park Rd, Wacol, QLD 4076 Australia; 3 Department of Psychology, Yale University, 2 Hillhouse Avenue- 208205, New Haven, CT 06520 USA; 4 Department of Psychiatry, University of British Columbia, UBC Hospital – Detwiller Pavilion, Room 2813, 2255 Wesbrook Mall, UBC Vancouver Campus, Vancouver, BC V6T 2A1 Canada; 5 Department of Global Health and Social Medicine, Harvard Medical School, 641 Huntington Avenue, Boston, MA 02115 USA; 6 Department of Psychiatry & Mental Health and South African Medical Council Research Unit on Risk and Resilience in Mental Disorders, University of Cape Town, Rondebosch, Cape Town, ZA 7925 South Africa; 7 Department of Social Medicine, Postgraduate Program in Public Health, Federal University of Espírito Santo, Espirito Santo – ES, Rua Dr. Euríco de Águiar, 888/705, Vitoria, Espirito Santo – ES, 2905-600 Brazil; 8 Center for Reducing Health Disparities, UC Davis Health System, 2921 Stockton Blvd., Suite 1408, Sacramento, CA 95817 USA; 9 College of Medicine, Al-Qadisiya University, P.O.Box 88, Al-Diwaniyah, Al-Qadisiyah, Iraq; 10 IMIM-Hospital del Mar Medical Research Institute, PRBB Building, Doctor Aiguader, 88, Barcelona, 08003 Spain; 11 CIBER en Epidemiología y Salud Pública (CIBERESP), Av. Monforte de Lemos, 3-5, Pabellón 11, Planta 0, Madrid, 28029 Spain; 12 Pompeu Fabra University (UPF), Plaça de la Mercè, 10-12, Barcelona, 08002 Spain; 13 University of São Paulo Medical School, Núcleo de Epidemiologia Psiquiátrica - LIM 23, Rua Dr. Ovidio Pires de Campos, 785, São Paulo, CEP 05403-010 Brazil; 14 School of Psychology, Ulster University, College Avenue, Londonderry, BT48 7JL UK; 15 Survey Research Center, Institute for Social Research, University of Michigan, 330 Packard, Room G358, Ann Arbor, MI 48104 USA; 16 Department of Psychiatry, University of Ibadan, University College Hospital, Ibadan, PMB 5116 Nigeria; 17 Shenzhen Institute of Mental Health & Shenzhen Kangning Hospital, Shenzhen, 518020 China; 18 Department of Health Care Policy, Harvard Medical School, 180 Longwood Avenue, Boston, MA 02115 USA; 19 Institute for Development, Research, Advocacy and Applied Care (IDRAAC), Achrafieh, St. George Hospital Street, Beirut, Lebanon; 20 Department of Psychiatry and Clinical Psychology, St George Hospital University Medical Center, Beirut, Ashrafieh 166378 Lebanon; 21 Faculty of Medicine, Balamand University, Rond Point Saloumeh, Sin el Fil, Beirut, Lebanon; 22 Unidad de Docencia, Investigacion y Formación en Salud Mental, Servicio Murciano de Salud, Murcia Health Service, C/ Lorca, nº 58. -El Palmar, Murcia, 30120 Spain; 23 Instituto Murciano de Investigación Biosanitaria Virgen de la Arrixaca, El Palmar, Murcia, 30120 Spain; 24 Centro de Investigación Biomédica en Red en Epidemíologia y Salud Pública, El Palmar, Murcia, 30120 Spain; 25 Department of Mental Health, Graduate School of Medicine, The University of Tokyo, 7-3-1, Hongo, Bunkyo-ku, Tokyo, 113-0033 Japan; 26 National Institute of Psychiatry Ramón de la Fuente Muñiz, Calz. Mexico-Xochimilco 101, San Lorenzo Huipulco, Ciudad de México, 14370 Mexico; 27 Department of Psychological Medicine, University of Otago, P.O. Box 56, Dunedin, 9054 New Zealand; 28 National Institute for Health Services Management, 31 Vaselor Str, Bucharest, 21253 Romania; 29 University Titu Maiorescu, Dâmbovnicului no. 22, Bucharest, Romania; 30 National Institute of Public Health, National Research Institute, 24 Chocimska St, Warsaw, 00-791 Poland; 31 Faculdade Ciências Médicas, Universidade Nova de Lisboa, Campo dos Mártires da Pátria, 130, Lisbon, 1169-056 Portugal; 32 Department of Mental Health, National Center of Public Health and Analyses, 15, Acad. Ivan Geshov Blvd, Sofia, 1431 Bulgaria

**Keywords:** Mental health services, Satisfaction, Perceived helpfulness, Patient perspectives, Healthcare providers, Mental disorders, Substance use disorders

## Abstract

**Background:**

Mental health service providers are increasingly interested in patient perspectives. We examined rates and predictors of patient-reported satisfaction and perceived helpfulness in a cross-national general population survey of adults with 12-month DSM-IV disorders who saw a provider for help with their mental health.

**Methods:**

Data were obtained from epidemiological surveys in the World Mental Health Survey Initiative. Respondents were asked about satisfaction with treatments received from up to 11 different types of providers (very satisfied, satisfied, neither satisfied nor dissatisfied, somewhat dissatisfied, very dissatisfied) and helpfulness of the provider (a lot, some, a little, not at all). We modelled predictors of satisfaction and helpfulness using a dataset of patient-provider observations (*n* = 5,248).

**Results:**

Most treatment was provided by general medical providers (37.4%), psychiatrists (18.4%) and psychologists (12.7%). Most patients were satisfied or very satisfied (65.9-87.5%, across provider) and helped a lot or some (64.4-90.3%). Spiritual advisors and healers were most often rated satisfactory and helpful. Social workers in human services settings were rated lowest on both dimensions. Patients also reported comparatively low satisfaction with general medical doctors and psychiatrists/psychologists and found general medical doctors less helpful than other providers. Men and students reported lower levels of satisfaction than women and nonstudents. Respondents with high education reported higher satisfaction and helpfulness than those with lower education. Type of mental disorder was unrelated to satisfaction but in some cases (depression, bipolar spectrum disorder, social phobia) was associated with low perceived helpfulness. Insurance was unrelated to either satisfaction or perceived helpfulness but in some cases was associated with elevated perceived helpfulness for a given level of satisfaction.

**Conclusions:**

Satisfaction with and perceived helpfulness of treatment varied as a function of type of provider, service setting, mental status, and socio-demographic variables. Invariably, caution is needed in combining data from multiple countries where there are cultural and service delivery variations. Even so, our findings underscore the utility of patient perspectives in treatment evaluation and may also be relevant in efforts to match patients to treatments.

**Supplementary Information:**

The online version contains supplementary material available at 10.1186/s13033-024-00629-7.

## Background

Mental health and substance use problems are leading causes of morbidity and premature mortality as well as of significant economic burden at both the individual and societal levels [1–3]. Despite the availability of potentially effective service models and interventions [4–7], there remains a significant gap between the number of people who need treatment for these problems and the number who receive it [8, 9]. Among those who receive treatment, moreover, not all patients are satisfied with the services they receive and not all find these services helpful. Increasingly, these patient perceptions are regarded as important considerations in mental health service delivery.

The concepts of satisfaction and perceived helpfulness are widely used to capture the patient perspective on treatment. Satisfaction is related to whether the services received are seen by the patient as adequate and delivered as expected. The process by which patients become satisfied or dissatisfied remains unclear. Prevailing theories suggest that satisfaction reflects the extent to which the patient’s expectations of treatment have been met or exceeded and that these expectations are largely determined by interpersonal aspects of care [10–13]. Despite the lack of clear conceptual underpinnings, patient reports of satisfaction are widely employed as indicators of health care quality [14]. Perceived helpfulness, in contrast, refers to the extent to which the patient attains personally meaningful goals through treatment. The two concepts both provide an evaluation of a health-care interaction with reference to concerns of importance to the patient, but we are not aware of previous research that has attempted to determine whether patterns and correlates of satisfaction and perceived helpfulness differ meaningfully depending on characteristics of the patient or provider.

Understanding patient perspectives may offer additional information about treatment gains over and above the information obtained from usual clinical outcomes (e.g., symptom reduction, daily functioning) [12, 15]. This is particularly relevant in the context of personalized (precision) treatment where the appropriate treatment may depend not only on symptom reduction but also on how the patient will perceive and view that treatment. Another important consideration is that satisfaction and perceived helpfulness might well relate to engagement or adherence with treatment recommendations [16, 17]. Failure to engage with or adhere to treatment recommendations, in turn, are associated with reduced quality of life and health outcomes and increased societal costs due to avoidable health care contacts [18, 19].

Recent epidemiological studies have advanced our knowledge about patterns and predictors of perceived helpfulness of mental health care in the real world based on analyses of large cross-national samples. These studies show that initial treatment contacts are often not helpful and that patients often have to see several providers before finding one that is perceived to be helpful [20]. Moreover, perceived helpfulness appears to vary according to treatment factors, such as the type(s) of providers seen and treatments received [20–22], as well as according to patient-level factors, such as age, socio-economic status, and mental disorder comorbidity [20–22]. As of yet, equivalent analyses have not considered satisfaction as an outcome. Nor have previous studies considered the relationship between satisfaction and perceived helpfulness. It could be that some types of care are associated with higher satisfaction but lower helpfulness and vice versa. Knowing if such differences exist might help increase our understanding of negative experiences of care [13].

The current report has three goals. The first is to describe levels of satisfaction and perceived helpfulness among patients who visited one or more providers for mental health problems in the previous 12 months. The second goal is to examine associations of socio-demographic, disorder, and treatment factors with between-patient variation in satisfaction and perceived helpfulness. The third goal is to determine whether these associations differ across the two perceptions; that is, whether some predictors are more important for satisfaction than for perceived helpfulness and vice versa. Participants came from 17 World Mental Health (WMH) surveys, a coordinated series of cross-national mental health needs assessment surveys carried out across countries in all major regions of the world [23].

## Methods

### Samples and procedures

Respondents were 18 years and older and came from 8 surveys in countries classified by the World Bank as low- or middle-income at the time of survey (Brazil, Bulgaria, Colombia – Medellin, Iraq, Mexico, People’s Republic of China – Shenzhen, Peru, and Romania) and 9 surveys in high-income countries (Argentina, New Zealand, Northern Ireland, Poland, Portugal, Saudi Arabia, Spain – Murcia, Japan, and United States). All surveys were based on multistage clustered area probability household samples. Nine of the surveys were nationally representative and the others were representative of selected regions, metropolitan areas, or urbanized areas. Response rates ranged from 50.4% (Poland) to 97.2% (Colombia – Medellin), with a weighted average across surveys of 69.4% (see Supplementary Table 1, Additional File 1).

Trained lay interviewers administered a fully structured diagnostic interview, the Composite International Diagnostic Interview Version 3.0 (CIDI 3.0) [24], face-to-face to respondents in their homes. The interview and training materials were developed in English and then translated into other languages following a standard translation protocol [25]. Interviewers were required to successfully complete a standardized training course before they could undertake fieldwork and collect data for this study. Consistent procedures were then used across surveys to check interviewer accuracy and ensure the use of consistent data cleaning and coding procedures [26]. Informed consent was obtained before starting the interview. Local institutional review committees approved and monitored the surveys to ensure protection of human subjects as per appropriate international and local guidelines.

The interview was split into two parts. Part I was administered to all respondents and assessed core mental disorders. Part II was administered to respondents who met lifetime criteria for any disorder in Part I plus a probability subsample of the remaining Part I respondents. Part II assessed additional disorders as well as correlates. Part II data were weighted to adjust for the under-sampling of Part I non-cases, thereby making the prevalence estimates of Part I disorders in the weighted Part II sample equivalent to prevalence in the Part I sample [27]. Of the 46,620 Part II respondents in the 17 surveys considered here, we focused on the 3,332 who met criteria for one or more of 8 disorders assessed in the CIDI (see next subsection) at some time in the 12 months before interview and who saw a provider for mental health problems at some time during that 12-month time period. As each of these respondents could have seen more than one type of provider, those who saw more than one type were counted as multiple observations (i.e., one observation for each respondent for each type of provider seen). This resulted in a total of 5,248 person-provider observations, which are the focus of the current report.

### Measures

Diagnoses: The CIDI assesses lifetime and 12-month disorders using the definitions and criteria of the *Diagnostic and Statistical Manual of Mental Disorders*, Fourth Edition (DSM-IV). Blinded clinical reappraisal studies have found good concordance between diagnoses based on the CIDI 3.0 and diagnoses based on blinded clinical gold standard diagnostic interviews with the Structured Clinical Interview for DSM-IV [28, 29]. As noted in the prior subsection, we consider here 8 12-month diagnostic categories: major depressive disorder, bipolar spectrum disorder, panic disorder/agoraphobia, generalized anxiety disorder, posttraumatic stress disorder, social phobia, specific phobia, and substance use disorders (alcohol and illicit drug abuse with or without dependence). DSM-IV organic exclusion rules were applied but diagnostic hierarchy rules were not applied other than between major depressive disorder and bipolar spectrum disorder.

Providers seen for mental health in the past year: All Part II respondents were asked if they had ever in their life seen each of a list of 11 different types of providers for problems with emotions, nerves, mental health, or use of alcohol or drugs and, if so, whether they had done so in the 12 months before interview. The list of providers was presented in a respondent booklet to assist with recall; examples of some types of providers were modified to fit the local context. The types of providers in the list were: general medical (including a general practitioner/primary care doctor, any other medical doctor other than a psychiatrist, and any other health care provider, such as a nurse or physician’s assistant other than a mental health provider); psychiatrist; other mental health professionals (psychologist; counsellor in a mental health specialized setting; social worker in a mental health specialized setting; any other mental health professional, such as a psychotherapist or mental health nurse); human services professionals (social worker in a human services setting; counsellor in a human services setting); and complementary/alternative medicine providers (spiritual advisor; any other type of healer).

Patient-reported satisfaction and helpfulness of treatment: For each type of provider seen in the 12 months before interview, respondents were asked additional questions that included two we focus on in the current report. (1) *‘In general, how satisfied are you with the treatments and services you received from the [TYPE OF PROFESSIONAL] in the past 12 months – very satisfied, satisfied, neither satisfied or dissatisfied, dissatisfied, or very dissatisfied?’* If more than one provider of that type was seen, the respondent was asked about the one they were most satisfied with. (2) *‘Did the [TYPE OF PROFESSIONAL] help you a lot, some, a little, or not at all?’* We created four dichotomous outcome variables so we could examine predictors of extent of satisfaction and perceived helpfulness: very satisfied (vs. all other categories); either satisfied or very satisfied (vs. all other categories); helped a lot (vs. all other categories); either helped some or a lot (vs. all other categories).

Predictors: The predictor of central interest was the type of provider seen. But we also considered three other types of predictors: socio-demographics, clinical factors, and treatment factors. Given the person-provider structure of the dataset, type of provider was represented as the provider type in each person-provider observation (dyad).

Socio-demographic predictors were gender, age in years (less than 35, 35–49, 50–64, 65 or over), marital status (married/cohabiting, never married, separated/widowed/divorced), employment (working, student, homemaker, retired, other), educational attainment and personal income (each coded into quartiles using country-specific coding schema). Clinical predictors were each of the eight 12-month mental disorder diagnoses. A variable representing number of mental disorders allowed us to capture effects of comorbidity. This is important because comorbidity may complicate diagnosis and treatment, and lead to greater functional impairment [30] and, in turn, impact the outcome of treatment. A variable representing the number of chronic physical conditions (exactly 1, exactly 2, 3 or more) was also included among the predictors because previous work has shown this to predict satisfaction with and perceived helpfulness of treatment for mental disorders [12, 15, 31].

Treatment-related predictors included type of health insurance (state-funded or subsidized, insurance through an employer or national social security, direct private/optional insurance, any other health insurance, no insurance coverage or unknown), which has been shown in some but not all studies to predict patient ratings of care [32, 33]. Other treatment-related predictors were the two helpfulness and two satisfaction variables, the first two of which we included in the final models to predict perceived satisfaction and the latter two of which we included in the final models to predict helpfulness. These final models allowed us to examine the extent to which the predictors of satisfaction differed from the predictors of helpfulness.

### Analysis methods

The analysis began by using simple cross-tabulations to examine the distribution of provider types seen and the associations between seeing one type of provider and seeing other types. We then used ordinary least squares (OLS) regression to examine associations of provider type with satisfaction and perceived helpfulness controlling for socio-demographics, other types of providers seen, clinical factors, and information about type of health insurance. In subsequent models we looked at discrepancies between predictors of satisfaction and predictors of perceived helpfulness by including information about one of these two variables as predictors in models for the other of the two variables. Logistic regression is typically used to estimate models of this type because OLS can generate individual-level predictions of outcome probabilities outside the 0-100% range when some predictors are continuous or models with discrete predictors are not saturated. This is not the case with logistic regression. However, the interpretations of logistic regression coefficients and of the odds-ratios obtained by exponentiating logistic regression coefficients are nonintuitive. Linear regression coefficients are more easily interpreted, as they represent differences in probabilities of the outcome associated with unit changes in the predictor. Furthermore, it can be shown that linear regression coefficients represent unbiased estimates of *average* causal effects of categorical predictors of the sort we consider here on probability of the dichotomous outcome when the conditions for making causal interpretations are met (i.e., values of the predictor are either randomized or are random with respect to other causes of the outcome and there is no informative loss to follow-up or informative measurement error) [34]. Based on these considerations, linear regression is used increasingly to estimate models of the sort we consider here [35].

The analyses were based on weighted data that adjusted for differential probabilities of selection as a function of selecting only one respondent per household regardless of the number of eligible respondents in the household (although this varied somewhat across studies) and also adjusted for deviation of the sample distribution from the known population distribution of socio-demographic and geographic variables. The statistical significance of regression coefficients was estimated using the Taylor-series linearization method [36], a design-based method that adjusted for this weighting as well as for the geographic clustering of the WMH data, to calculate 95% confidence intervals of the regression coefficients. The significance of sets of coefficients defining a single categorical variable (e.g., dummy variables defining respondent marital status) and the full set of coefficients was evaluated with Wald F tests based on design-corrected coefficient variance − covariance matrices. Statistical significance was evaluated consistently using two-sided design based 0.05-level tests. All analyses were implemented in SAS 9.4 [37].

## Results

Of the 46,620 Part II respondents, 13.9% (*n* = 10,518) met criteria for one of the 8 12-month mental disorders included here. Of these, 30.3% (*n* = 3,332) respondents reported that they saw a service provider for their mental health in the 12 months before interview (see Supplementary Table 1, Additional File 1). Rates varied significantly across countries, from 6.9% in Shenzhen to 50.6% in Northern Ireland (*p* < 0.001). Respondents with a 12-month disorder in high-income countries were, on average, twice as likely to have seen a provider in the past 12 months as those in low/middle-income countries (35.7% vs. 17.4%, *p* < 0.001).

### Distribution of treatment across types of providers

These 3,332 patients provided a total of 5,248 patient-provider observations for analysis. This means that we took into consideration the fact that some of them saw multiple types of providers (Table 1). The providers seen most commonly were general medical providers, who were seen by 59.3% of all patients (column A). Thinking of the patient-provider dyad as the unit of analysis, 37.4% of such dyads were with general medical providers (column B). The next commonly seen providers were psychiatrists and psychologists. The least commonly seen were social workers in a human services setting, counsellors in a human services setting, and other health professionals.

**Table 1:** Types of providers seen among respondents who reported 12-month use of providers for mental health and have at least one disorder

	A: Proportion of patients^a^			B: Proportion of patient-provider observations^b^			C: Proportion of people who saw at least one other provider^c^			D: Mean number of the other providers seen	
	%	(SE)		%	(SE)		%	(SE)		Mean	(SD)
I. **General medical**											
Doctor	59.3	(0.9)		37.4	(0.7)		44.1	(1.4)		2.7	(1.1)
Other health professional	3.9	(0.4)		2.5	(0.2)		83.5	(2.0)		3.8	(1.8)
II. **Specialty mental health**											
Psychiatrist	29.1	(0.9)		18.4	(0.5)		64.0	(1.6)		2.9	(1.2)
Psychologist	20.2	(0.9)		12.7	(0.6)		68.3	(1.9)		2.9	(1.3)
Counsellor in a mental health specialized setting	12.6	(0.7)		7.9	(0.4)		72.4	(1.8)		3.1	(1.4)
Social worker in a mental health specialized setting	5.5	(0.4)		3.3	(0.2)		84.7	(1.9)		3.7	(1.7)
Other mental health professional	5.3	(0.5)		3.3	(0.3)		87.7	(1.8)		3.6	(1.7)
III. **Human services**											
Social worker in a human services setting	1.7	(0.2)		1.1	(0.2)		87.6	(0.0)		3.9	(2.0)
Counsellor in a human services setting	2.5	(0.3)		1.5	(0.2)		76.2	(1.1)		3.5	(1.6)
IV. **Complementary and alternative medicine**											
Spiritual advisor	12.2	(0.8)		7.7	(0.5)		57.4	(2.3)		3.1	(1.4)
Healer	6.5	(0.6)		4.1	(0.3)		68.9	(1.6)		3.1	(1.5)
V. **Total**	100	-		100.0	-		36.4	(1.1)		2.6	(1.0)
(n)	(3,332)			(5,248)							

^a^ Respondents who reported any 12-month use of providers for mental health

^b^ Person-provider observations, where respondents who saw more than one type of provider were counted as multiple observations

^c^ Indicates the extent to which treatment by a given provider occurred in combination with another provider. A higher percentage indicates a greater proportion of treatment in combination

Some sense of the overlap among types of providers is shown in column C of Table 1, where we see that 36.4% of patients saw two or more types of providers, with a mean of 3.6 provider types among those who saw two or more types. The probability of seeing multiple provider types was highest for those who saw other mental health professionals or social workers in human services sectors (87.6–87.7%) and lowest for those who saw a general medical practitioner (44.1%).

### Patient ratings of satisfaction and helpfulness for different types of providers

Table 2 presents distributions of patient ratings of satisfaction and perceived helpfulness along with the cross-classification of the two ratings. Ratings of being very satisfied varied more than two-fold, from a high of over half of patients who saw a healer or spiritual advisor (56.8–57.2%) to a low of 19.0% among those who saw a social worker in a human services setting. There was relatively less variability across providers in the proportion of patients who said they were at least satisfied (i.e., gave a rating of very satisfied or satisfied), with most patients endorsing these responses (65.9-87.5%). At the other end of the spectrum, only a minority of patients said they were dissatisfied or very dissatisfied, with the highest proportion among patients who saw a psychiatrist (14.0%) and the lowest among patients who saw a spiritual advisor, other mental health specialist or healer (3.2-4.5%). Ratings of being helped a lot varied in a similar way across the different types of providers, from 63.5 to 68.2% among those who saw a healer or spiritual advisor to 31.6% among patients who saw a social worker in a human services setting. Across all types of providers, most patients said they were either helped a lot or some (64.4-90.3%). Few patients said they were not helped at all, with the highest proportion being among those who saw a social worker in a human services setting (19.1%) and the lowest among patients who saw a spiritual advisor (2.3%).

**Table 2:** Distribution of patient-reported satisfaction by helpfulness for each type of provider seen, regardless of other providers seen (*n* = 5,248)

Type of provider	Satisfaction	Helpfulness												Correlation between satisfaction and helpfulness
		A lot			Some			A little			Not at all			
		%	(SE)		%	(SE)		%	(SE)		%	(SE)		r
I. **General medical**														
Doctor	Very satisfied	31.8	(1.1)		3.3	(0.5)		0.9	(0.2)		0.3	(0.1)		0.72
	Satisfied	14.0	(0.8)		15.7	(1.0)		3.9	(0.5)		0.5	(0.1)		
	Neither satisfied or dissatisfied	1.1	(0.2)		6.2	(0.6)		6.6	(0.7)		2.7	(0.4)		
	Dissatisfied/Very dissatisfied	0.6	(0.3)		1.5	(0.3)		4.5	(0.5)		6.3	(0.7)		
Other health professional	Very satisfied	37.8	(4.1)		0.4	(0.4)		1.4	(0.1)		0.0	(0.0)		0.84
	Satisfied	8.5	(1.9)		25.6	(3.8)		2.0	(0.1)		1.1	(0.1)		
	Neither satisfied or dissatisfied	0.0	(0.0)		4.7	(1.1)		7.2	(1.0)		3.3	(2.2)		
	Dissatisfied/Very dissatisfied	0.0	(0.0)		1.0	(0.1)		0.2	(0.0)		6.8	(0.7)		
II. **Specialty mental health**														
Psychiatrist	Very satisfied	30.1	(1.6)		2.0	(0.6)		0.2	(0.1)		0.2	(0.2)		0.75
	Satisfied	16.6	(1.4)		14.3	(1.1)		3.6	(0.7)		0.4	(0.2)		
	Neither satisfied or dissatisfied	1.8	(0.5)		6.6	(0.9)		7.1	(0.9)		3.1	(0.6)		
	Dissatisfied/Very dissatisfied	0.4	(0.2)		1.3	(0.5)		4.9	(0.8)		7.4	(1.1)		
Psychologist	Very satisfied	30.9	(1.5)		1.5	(0.5)		0.2	(0.1)		0.0	(0.0)		0.80
	Satisfied	16.3	(1.3)		18.3	(1.4)		4.4	(0.4)		0.1	(0.1)		
	Neither satisfied or dissatisfied	0.5	(0.2)		6.5	(0.8)		6.2	(0.8)		3.5	(0.9)		
	Dissatisfied/Very dissatisfied	0.2	(0.1)		1.1	(0.3)		2.4	(0.7)		8.1	(0.9)		
Counsellor in a mental health specialized setting	Very satisfied	37.1	(2.5)		3.9	(0.7)		0.1	(0.1)		0.3	(0.3)		0.75
	Satisfied	16.3	(2.2)		21.2	(1.9)		3.0	(0.8)		0.0	(0.0)		
	Neither satisfied or dissatisfied	0.0	(0.0)		4.8	(0.8)		4.7	(0.9)		1.4	(0.4)		
	Dissatisfied/Very dissatisfied	0.4	(0.3)		0.4	(0.3)		1.6	(0.6)		4.7	(1.0)		
Social worker in a mental health specialized setting	Very satisfied	31.0	(3.0)		4.7	(2.0)		3.8	(0.1)		0.0	(0.0)		0.78
	Satisfied	12.3	(2.2)		21.4	(1.9)		4.7	(0.9)		0.7	(0.0)		
	Neither satisfied or dissatisfied	0.5	(0.4)		4.6	(0.8)		5.2	(0.9)		1.0	(1.0)		
	Dissatisfied/Very dissatisfied	0.6	(0.0)		0.0	(0.0)		2.0	(0.4)		7.5	(0.9)		
Other mental health professional	Very satisfied	37.0	(2.4)		1.5	(0.4)		1.2	(0.1)		0.0	(0.0)		0.78
	Satisfied	9.9	(1.1)		18.5	(3.3)		2.3	(0.1)		0.5	(0.0)		
	Neither satisfied or dissatisfied	0.8	(0.1)		3.3	(0.5)		15.8	(2.8)		4.9	(1.1)		
	Dissatisfied/Very dissatisfied	0.0	(0.0)		0.0	(0.0)		1.9	(0.7)		2.3	(1.0)		
III. **Human services**														
Social worker in a human services setting	Very satisfied	16.1	(0.2)		2.9	(1.2)		0.0	(0.0)		0.0	(0.0)		0.68
	Satisfied	15.5	(0.0)		19.4	(0.1)		12.0	(1.3)		0.0	(0.0)		
	Neither satisfied or dissatisfied	0.0	(0.0)		7.0	(0.0)		2.6	(0.0)		13.1	(0.0)		
	Dissatisfied/Very dissatisfied	0.0	(0.0)		3.5	(0.0)		1.9	(0.0)		6.0	(0.0)		
Counsellor in a human services setting	Very satisfied	33.3	(2.2)		1.6	(0.0)		2.1	(0.0)		0.0	(0.0)		0.70
	Satisfied	24.0	(2.6)		15.7	(1.8)		2.3	(0.1)		2.4	(0.8)		
	Neither satisfied or dissatisfied	0.0	(0.0)		5.8	(0.1)		1.1	(0.8)		3.9	(1.4)		
	Dissatisfied/Very dissatisfied	0.0	(0.0)		0.0	(0.0)		2.7	(0.1)		5.1	(1.7)		
IV. **Complementary and alternative medicine**														
Spiritual advisor	Very satisfied	50.8	(2.1)		5.4	(1.8)		1.0	(0.4)		0.0	(0.0)		0.68
	Satisfied	16.2	(1.9)		11.7	(1.2)		1.8	(0.4)		0.6	(0.4)		
	Neither satisfied or dissatisfied	0.7	(0.3)		4.1	(0.6)		4.0	(0.5)		0.5	(0.2)		
	Dissatisfied/Very dissatisfied	0.5	(0.0)		0.9	(0.4)		0.6	(0.1)		1.2	(0.3)		
Healer	Very satisfied	54.0	(1.7)		2.5	(0.5)		0.0	(0.0)		0.3	(0.0)		0.81
	Satisfied	8.8	(0.7)		13.8	(1.9)		1.5	(0.5)		0.7	(0.0)		
	Neither satisfied or dissatisfied	0.7	(0.0)		3.4	(0.1)		7.6	(0.4)		2.2	(0.4)		
	Dissatisfied/Very dissatisfied	0.0	(0.0)		0.5	(0.0)		0.5	(0.0)		3.5	(0.8)		

Patient ratings of satisfaction and helpfulness showed moderate-to-strong correlations, with the highest being for healers, psychologists and other health professionals (*r* = 0.80–0.84) and the lowest being for social workers in a human services setting and spiritual advisors (*r* = 0.68). Correlations clustered in the range *r* = 0.75–0.80 for the different types of mental health specialty providers.

### Associations of predictors with satisfaction and helpfulness

Regression models predicting the two satisfaction outcomes – very satisfied and either very satisfied or satisfied – are shown in Table 3. Probabilities of being satisfied (F_10,634_=8.1, *p* < 0.001) and very satisfied (F_10,634_=7.4, *p* < 0.001) both varied significantly across provider type. Ipsative coding was used for providers, which means that all 11 types of providers were compared to the average. In both models, patients were most likely to be satisfied with spiritual advisors. Patients were also significantly more likely to be very satisfied with healers and more likely to be satisfied with counsellors in a mental health specialty setting than other providers. Patients were least likely to be very satisfied with social workers in a human services setting and also less likely to be satisfied (either very or somewhat) with general medical doctors and psychiatrists than other types of providers.

**Table 3:** Joint associations of provider, sociodemographic, clinical and treatment predictors with patient-reported satisfaction outcomes, controlling for helpfulness, across all providers seen by respondents who reported 12-month use of providers for mental health (*n* = 5,248)a

	Very satisfied								Either satisfied or very satisfied						
	Base model				Model with helpfulness controls				Base model				Model with helpfulness controls		
	Estimate	95%CI lower	95%CI upper		Estimate	95%CI lower	95%CI upper		Estimate	95%CI lower	95%CI upper		Estimate	95%CI lower	95%CI upper
**I. Type of provider**:															
A. **General medical**															
Doctor	-0.05*	-0.08	-0.01		-0.01	-0.04	0.02		-0.07*	-0.10	-0.04		-0.03*	-0.06	-0.01
Other health professional	0.01	-0.09	0.11		0.03	-0.04	0.09		0.03	-0.04	0.11		0.03	-0.03	0.09
B. **Specialty mental health**															
Psychiatrist	-0.05*	-0.09	-0.01		-0.04*	-0.08	-0.00		-0.08*	-0.13	-0.04		-0.06*	-0.09	-0.03
Psychologist	-0.05*	-0.10	0.00		-0.03	-0.06	0.01		-0.05	-0.10	0.00		-0.03	-0.06	0.01
Counsellor in a mental health specialized setting	0.01	-0.04	0.07		-0.01	-0.05	0.04		0.05*	0.01	0.10		0.01	-0.02	0.05
Social worker in a mental health specialized setting	0.01	-0.07	0.09		0.04	-0.03	0.11		0.04	-0.02	0.11		0.06*	0.00	0.12
Other mental health professional	-0.01	-0.08	0.06		0.01	-0.04	0.06		-0.06	-0.13	0.02		-0.01	-0.06	0.04
C. **Human services**															
Social worker in a human services setting	-0.17*	-0.25	-0.09		-0.09*	-0.17	-0.01		-0.06	-0.20	0.07		0.02	-0.09	0.12
Counsellor in a human services setting	-0.03	-0.13	0.08		-0.07	-0.17	0.03		0.06	-0.03	0.15		0.03	-0.04	0.09
D. **Complementary and alternative medicine**															
Spiritual advisor	0.16*	0.11	0.22		0.06*	0.01	0.11		0.09*	0.05	0.13		-0.02	-0.05	0.02
Healer	0.16*	0.08	0.24		0.10*	0.05	0.16		0.04	-0.02	0.11		0.00	-0.04	0.03
F-test: F_10,634_ (p-value)	7.38* (< 0.001)				3.28* (< 0.001)				8.12* (< 0.001)				2.52* (0.006)		
**II. Gender**:															
Male	-0.06*	-0.12	-0.01		-0.03	-0.07	0.00		-0.05*	-0.09	-0.00		-0.01	-0.04	0.02
Female	ref.				ref.				ref.				ref.		
F-test: F_1,634_ (p-value)	5.73* (0.02)				3.59 (0.06)				4.45* (0.035)				0.39 (0.53)		
**III. Age at interview (years)**:															
Less than 35	-0.04	-0.14	0.06		0.01	-0.06	0.08		-0.05	-0.13	0.03		-0.01	-0.07	0.05
35–49	-0.05	-0.14	0.04		-0.01	-0.08	0.06		-0.05	-0.12	0.02		-0.02	-0.08	0.04
50–64	0.02	-0.07	0.11		0.01	-0.05	0.08		0.02	-0.05	0.08		0.00	-0.05	0.06
65 or over	ref.				ref.				ref.				ref.		
F-test: F_3,634_ (p-value)	2.74 (0.061)				0.82 (0.48)				3.96 (0.056)				1.02 (0.38)		
**IV. Marital status**:															
Married/cohabitating	ref.				ref.				ref.				ref.		
Never married	0.00	-0.06	0.06		-0.01	-0.05	0.03		0.01	-0.04	0.05		-0.01	-0.03	0.02
Separated/widowed/divorced	-0.03	-0.07	0.02		-0.03	-0.06	0.01		0.00	-0.05	0.05		0.00	-0.03	0.04
F-test: F_3,634_ (p-value)	0.78 (0.46)				0.94 (0.39)				0.04 (0.95)				0.11 (0.89)		
**V. Employment**:															
Homemaker	-0.04	-0.09	0.02		-0.02	-0.06	0.03		-0.02	-0.07	0.03		0.02	-0.03	0.06
Retired	-0.02	-0.12	0.08		0.00	-0.08	0.07		0.01	-0.07	0.10		0.01	-0.06	0.08
Student	-0.14*	-0.22	-0.05		-0.09*	-0.18	-0.00		-0.09*	-0.17	-0.01		-0.04	-0.09	0.02
Other	-0.01	-0.07	0.04		0.01	-0.03	0.05		-0.03	-0.08	0.02		0.00	-0.03	0.03
Working	ref.				ref.				ref.				ref.		
F-tests: F_4,634_ (p-value)	2.93* (0.02)				1.50 (0.20)				1.73 (0.14)				0.75 (0.56)		
**VI. Education**^**b**^:															
Low	-0.09*	-0.15	-0.02		-0.05*	-0.11	-0.00		-0.06*	-0.12	-0.01		-0.03	-0.07	0.01
Low-average	-0.05	-0.11	0.00		-0.03	-0.07	0.01		-0.06*	-0.11	-0.01		-0.04*	-0.07	-0.00
High-average	-0.08*	-0.13	-0.03		-0.03	-0.07	0.00		-0.08*	-0.12	-0.04		-0.04*	-0.07	-0.01
High	ref.				ref.				ref.				ref.		
F-test: F_3,634_ (p-value)	4.25* (0.001)				1.77 (0.15)				5.92* (0.001)				3.05* (0.03)		
**VII. 12-month mental disorders**:															
Major depressive disorder	-0.05*	-0.11	0.00		-0.01	-0.05	0.04		-0.05*	-0.09	-0.00		0.00	-0.03	0.02
Bipolar spectrum disorder	-0.08*	-0.15	-0.01		-0.02	-0.08	0.03		-0.04	-0.10	0.03		0.01	-0.03	0.05
Generalized anxiety disorder	-0.04	-0.10	0.02		-0.02	-0.06	0.02		-0.01	-0.07	0.04		0.01	-0.03	0.05
Panic disorder/Agoraphobia	-0.04	-0.10	0.02		-0.03	-0.07	0.01		0.00	-0.06	0.05		0.01	-0.03	0.04
Posttraumatic stress disorder	-0.05	-0.11	0.01		-0.03	-0.08	0.02		-0.03	-0.09	0.02		-0.01	-0.04	0.03
Specific phobia	0.01	-0.04	0.06		0.00	-0.04	0.04		-0.01	-0.05	0.04		-0.01	-0.04	0.02
Social phobia	-0.05	-0.10	0.01		0.00	-0.04	0.04		-0.03	-0.08	0.03		0.00	-0.04	0.04
Substance use disorder	-0.02	-0.08	0.05		0.01	-0.04	0.06		-0.02	-0.09	0.04		0.01	-0.03	0.05
F-test: F_7,634_ (p-value)	1.76 (0.09)				0.73 (0.64)				0.45 (0.87)				0.28 (0.96)		
F-test: F_8,634_ (p-value)	1.65 (0.11)				0.69 (0.70)				0.86 (0.55)				0.29 (0.97)		
**VIII. Number of 12-month mental disorders**:															
1 or 2	ref.				ref.				ref.				ref.		
3 or more	0.05	-0.03	0.13		0.02	-0.04	0.09		-0.03	-0.11	0.05		-0.05	-0.10	0.00
F-test: F_1,634_ (p-value)	1.45 (0.23)				0.58 (0.45)				0.54 (0.46)				3.73 (0.05)		
**IX. Number of chronic physical disorders**^**c**^:															
0 or 1	ref.				ref.				ref.				ref.		
2	-0.01	-0.05	0.03		0.00	-0.04	0.04		-0.01	-0.05	0.03		0.00	-0.03	0.03
3 or more	0.03	-0.03	0.08		0.02	-0.02	0.05		-0.02	-0.07	0.02		-0.01	-0.04	0.02
F-test: F_2,634_ (p-value)	0.85 (0.43)				0.47 (0.63)				0.62 (0.54)				0.40 (0.67)		
**X. Insurance**:															
None or unknown	ref.				ref.				ref.				ref.		
State funded coverage or subsidized insurance	0.02	-0.10	0.15		0.00	-0.10	0.09		0.09*	0.00	0.18		0.04	-0.02	0.10
Other	0.04	-0.08	0.16		0.01	-0.09	0.12		0.08*	0.00	0.16		0.02	-0.03	0.08
Insurance through employment or national social security	0.06	-0.06	0.17		0.03	-0.07	0.13		0.09*	0.00	0.17		0.05	-0.01	0.12
Direct private/optional insurance	-0.04	-0.17	0.10		-0.02	-0.12	0.07		0.10	-0.06	0.26		0.03	-0.09	0.15
F-test: F_4,634_ (p-value)	0.53 (0.72)				0.66 (0.62)				1.44 (0.22)				0.86 (0.49)		
**XI. Helped a lot**:															
Yes					0.56*	0.52	0.60						0.24*	0.20	0.27
No					ref.								ref.		
F-test: F_1,634_ (p-value)	n/a				897.7* (< 0.0001)				n/a				219.1* (< 0.0001)		
**XII. Either helped a lot or some**:															
Yes					0.06*	0.03	0.09						0.52*	0.47	0.56
No					ref.								ref.		
F-test: F_1,634_(p-value)	n/a				14.3* (< 0.0002)				n/a				547.4* (< 0.0001)		

Abbreviations: n/a, not included in model. ref., reference category. * Significant at 0.05 level, two-sided test

^a^ Results from ordinary least squares regression models that included survey dummy variables

^b^ Country-specific distributions. See Evans-Lacko et al. (2018) for details

^c^ Chronic physical disorders included were: Arthritis; Cancer; Cardiovascular disorders (heart attack, heart disease, hypertension and stroke); Chronic pain conditions (chronic back or neck pain and other chronic pain); Diabetes; Migraine or other frequent or severe headaches; Neurological disorders (multiple sclerosis, Parkinson’s and epilepsy or seizures); Digestive disorders (stomach or intestinal ulcers and irritable bowel disorder); Respiratory disorders (seasonal allergies, asthma, chronic obstructive pulmonary disease and emphysema)

Pooled across types of providers, men were less likely to be satisfied (either very or somewhat) than women, students less likely than nonstudents, and respondents with all but the highest level of education less likely to be very or somewhat satisfied than those with the highest level of education. The other socio-demographic variables, age and marital status, in comparison, were unrelated to patient satisfaction. Number/type of mental disorders and physical disorders and health insurance were also unrelated to patient satisfaction.

We then added controls for helpfulness to the models predicting satisfaction (Table 3). When this was done, only the associations with type of provider and, in the case of somewhat satisfied, education remained significant. Most notably, patients seen by spiritual advisors and healers were more likely relative to other patients with the same levels of perceived helpfulness to report being very satisfied, whereas patients seen by psychiatrists were less likely than other patients with the same levels of perceived helpfulness to report being either very or somewhat satisfied. Patients with the highest level of education were significantly more likely to be somewhat satisfied than those with less education at the same levels of perceived helpfulness.

Equivalent models for the two helpfulness outcomes – helped a lot and either helped some or a lot – are shown in Table 4. As with satisfaction, perceived helpfulness varied significantly with type of provider (F_10,634_=7.5, *p* < 0.001 for helped either some or a lot; F_10,634_=6.9, *p* < 0.001 for being helped a lot). In the base models, patients were most likely to report being helped either a lot or somewhat by spiritual advisors and to have higher probabilities of being helped a lot by healers and helped somewhat by counsellors in a mental health specialty setting. Patients were least likely to perceive social workers in a human services setting as very helpful and were also significantly less likely to perceive general medical doctors than other providers as either very or somewhat helpful.

**Table 4:** Joint associations of provider, sociodemographic, clinical and treatment factors with patient-reported helpfulness outcomes, controlling for satisfaction, among respondents who reported 12-month use of providers for mental health (*n* = 5,248)a

	Helped a lot								Either helped a lot or some						
	Base model				Model with satisfaction controls				Base model				Model with satisfaction controls		
	Estimate	95%CI lower	95%CI upper		Estimate	95%CI lower	95%CI upper		Estimate	95%CI lower	95%CI upper		Estimate	95%CI lower	95%CI upper
**I. Type of provider**:															
E. **General medical**															
Doctor	-0.06*	-0.10	-0.02		-0.01	-0.04	0.02		-0.05*	-0.08	-0.01		0.00	-0.03	0.03
Other health professional	-0.03	-0.14	0.07		-0.05	-0.12	0.02		0.02	-0.04	0.09		0.00	-0.05	0.05
F. **Specialty mental health**															
Psychiatrist	-0.01	-0.06	0.03		0.04*	0.01	0.08		-0.04	-0.08	0.00		0.01	-0.02	0.05
Psychologist	-0.04	-0.09	0.01		0.00	-0.03	0.04		-0.03	-0.07	0.02		0.01	-0.03	0.04
Counsellor in a mental health specialized setting	0.03	-0.03	0.09		0.01	-0.04	0.05		0.06*	0.02	0.10		0.03	0.00	0.06
Social worker in a mental health specialized setting	-0.05	-0.12	0.02		-0.07*	-0.14	-0.01		-0.01	-0.09	0.07		-0.03	-0.11	0.04
Other mental health professional	-0.04	-0.11	0.03		-0.01	-0.06	0.04		-0.07*	-0.14	-0.00		-0.04	-0.08	0.01
G. **Human services**															
Social worker in a human services setting	-0.14*	-0.25	-0.02		-0.03	-0.13	0.06		-0.09	-0.23	0.04		-0.04	-0.15	0.07
Counsellor in a human services setting	0.07	-0.06	0.20		0.06	-0.04	0.17		0.03	-0.07	0.13		0.00	-0.08	0.08
H. **Complementary and alternative medicine**															
Spiritual advisor	0.17*	0.11	0.23		0.06*	0.01	0.11		0.12*	0.08	0.16		0.05*	0.02	0.09
Healer	0.10*	0.01	0.18		0.00	-0.04	0.05		0.05	-0.02	0.12		0.01	-0.03	0.05
F-test: F_10,634_(p-value)	6.94* (< 0.001)				2.71* (0.003)				7.55* (< 0.001)				1.69 (0.079)		
**II. Gender**:															
Male	-0.05	-0.10	0.00		0.00	-0.03	0.03		-0.05	-0.09	-0.01*		-0.02	-0.04	0.01
Female	ref.				ref.				ref.				ref.		
F-test: F_1,634_ (p-value)	3.64 (0.06)				0.00 (0.95)				6.19* (0.013)				1.76 (0.19)		
**III. Age at interview (years)**:															
Less than 35	-0.09	-0.21	0.03		-0.05	-0.14	0.03		-0.04	-0.13	-0.05		-0.01	-0.08	0.07
35–49	-0.07	-0.17	0.03		-0.02	-0.10	0.05		-0.03	-0.12	0.05		0.00	-0.07	0.07
50–64	0.02	-0.08	0.11		0.00	-0.07	0.07		0.02	-0.06	0.10		0.01	-0.05	0.07
65 or over	ref.				ref.				ref.				ref.		
F-test: F_3,634_ (p-value)	3.85 (0.07)				1.44 (0.23)				2.62 (0.051)				0.15 (0.93)		
**IV. Marital status**:															
Married/cohabitating	ref.				ref.				ref.				ref.		
Never married	0.02	-0.03	0.07		0.01	-0.02	0.04		0.02	-0.03	0.06		0.01	-0.02	0.04
Separated/widowed/divorced	0.00	-0.05	0.05		0.01	-0.03	0.05		0.00	-0.05	0.05		0.00	-0.03	0.04
F-test: F_2,634_ (p-value)	0.25 (0.78)				0.42 (0.67)				0.31 (0.73)				0.37 (0.69)		
**V. Employment**															
Homemaker	-0.03	-0.09	0.03		-0.01	-0.05	0.04		-0.06*	-0.11	-0.01		-0.04*	-0.09	-0.00
Retired	-0.03	-0.12	0.07		-0.03	-0.09	0.04		0.02	-0.05	0.08		0.01	-0.05	0.07
Student	-0.07	-0.17	0.02		0.02	-0.06	0.11		-0.07	-0.14	0.01		0.00	-0.05	0.05
Other	-0.04	-0.10	0.01		-0.03	-0.06	0.01		-0.03	-0.09	0.02		-0.02	-0.05	0.02
Working	ref.				ref.				ref.				ref.		
F-test: F_4,634_ (p-value)	1.29 (0.27)				0.84 (0.50)				2.71* (0.03)				1.44 (0.22)		
**VI. Education**^**b**^:															
Low	-0.05	-0.12	0.01		0.01	-0.03	0.06		-0.04	-0.09	0.02		0.01	-0.03	0.04
Low-average	-0.04	-0.09	0.01		0.01	-0.03	0.05		-0.03	-0.08	0.02		0.01	-0.02	0.04
High-average	-0.08*	-0.13	-0.03		-0.01	-0.05	0.03		-0.05*	-0.09	-0.00		0.01	-0.02	0.04
High	ref.				ref.				ref.				ref.		
F-test: F_3,634_ (p-value)	3.58* (0.014)				0.48 (0.70)				1.80 (0.15)				0.15 (0.93)		
**VII. 12-month mental disorders**:															
Major depressive disorder	-0.08*	-0.13	-0.02		-0.03	-0.07	0.01		-0.05*	-0.09	-0.00		-0.02	-0.05	0.01
Bipolar spectrum disorder	-0.10*	-0.17	-0.03		-0.05*	-0.09	-0.00		-0.04	-0.10	0.01		-0.02	-0.05	0.02
Generalized anxiety disorder	-0.03	-0.10	0.03		-0.01	-0.05	0.03		-0.03	-0.09	0.02		-0.02	-0.07	0.02
Panic disorder/Agoraphobia	-0.01	-0.07	0.05		0.01	-0.02	0.04		-0.01	-0.06	0.04		-0.01	-0.04	0.03
Posttraumatic stress disorder	-0.04	-0.10	0.01		0.00	-0.05	0.04		-0.04	-0.08	0.01		-0.01	-0.05	0.02
Specific phobia	0.02	-0.03	0.07		0.02	-0.01	0.05		-0.01	-0.05	0.03		-0.01	-0.04	0.02
Social phobia	-0.07*	-0.13	-0.02		-0.04*	-0.08	-0.00		-0.02	-0.07	0.03		0.00	-0.03	0.03
Substance use disorder	-0.02	-0.09	0.05		-0.01	-0.05	0.03		-0.06	-0.12	0.00		-0.04*	-0.09	-0.00
F-test: F_7,634_ (p-value)	3.01* (0.004)				2.24* (0.03)				1.02 (0.41)				0.84 (0.56)		
F-test: F_8,634_ (p-value)	3.39* (< 0.001)				2.27* (0.02)				1.17 (0.31)				0.75 (0.65)		
**VIII. Number of 12-month mental disorders**:															
1 or 2	ref.				ref.				ref.				ref.		
3 or more	0.04	-0.04	0.13		0.03	-0.03	0.09		0.02	-0.05	0.09		0.03	-0.02	0.08
F-test: F_1,634_ (p-value)	1.04 (0.31)				1.23 (0.27)				0.29 (0.59)				1.64 (0.20)		
**IX. Number of chronic physical disorders**^**c**^:															
0 or 1	ref.				ref.				ref.				ref.		
2	-0.02	-0.06	0.03		-0.01	-0.05	0.03		-0.02	-0.06	0.02		-0.01	-0.04	0.02
3 or more	0.02	-0.03	0.07		0.02	-0.01	0.05		-0.03	-0.08	0.01		-0.02	-0.05	0.01
F-test: F_2,634_ (p-value)	0.91 (0.40)				1.18 (0.31)				1.35 (0.26)				1.18 (0.31)		
**X. Insurance**:															
None or unknown	ref.				ref.				ref.				ref.		
State funded coverage or subsidized insurance	0.04	-0.05	0.13		0.00	-0.07	0.06		0.07	-0.02	0.16		0.01	-0.04	0.07
Other	0.03	-0.05	0.11		-0.02	-0.09	0.05		0.10*	0.02	0.18		0.05	0.00	0.10
Insurance through employment or national social security	0.04	-0.05	0.12		-0.02	-0.09	0.05		0.05	-0.03	0.14		0.00	-0.06	0.06
Direct private/optional insurance	-0.04	-0.20	0.11		-0.06	-0.17	0.05		0.16*	0.05	0.27		0.10*	0.01	0.19
F-test: F_4,634_ (p-value)	0.60 (0.66)				0.60 (0.66)				2.97* (0.02)				2.75* (0.03)		
**XI. Very satisfied**:															
Yes					0.47*	0.44	0.51						0.09*	0.06	0.11
No					ref.								ref.		
F-test: F_1,634_(p-value)	n/a				720.2* (< 0.001)				n/a				60.9* (< 0.001)		
**XII. Either satisfied or very satisfied**:															
Yes					0.37*	0.34	0.40						0.58*	0.54	0.62
No					ref.								ref.		
F-test: F_1,634_(p-value)	n/a				604.1* (< 0.001)				n/a				896.8* (< 0.001)		

Abbreviations: n/a, not included in model. ref., reference category.* Significant at 0.05 level, two-sided test

^a^ Results from ordinary least squares regression models that included survey dummy variables

^b^ Country-specific distributions. See Evans-Lacko et al. (2018) for details

^c^ Chronic physical disorders included were: Arthritis; Cancer; Cardiovascular disorders (heart attack, heart disease, hypertension and stroke); Chronic pain conditions (chronic back or neck pain and other chronic pain); Diabetes; Migraine or other frequent or severe headaches; Neurological disorders (multiple sclerosis, Parkinson’s and epilepsy or seizures); Digestive disorders (stomach or intestinal ulcers and irritable bowel disorder); Respiratory disorders (seasonal allergies, asthma, chronic obstructive pulmonary disease and emphysema)

The perception of being helped a lot was positively associated with only one socio-demographic variable, high education, whereas the perception of being helped somewhat was lower among men and homemakers than others. Mental disorders were significant as a set in predicting the perception of being helped a lot but not somewhat, with significant variation across disorders due to depression, bipolar spectrum disorder, and social phobia associated with a low probability of the perception of being helped a lot. Physical disorders were unrelated to perceived helpfulness (either a lot or some). Insurance, in comparison, most notably direct private/optional insurance and other insurance, was associated with significantly increased probability of the perception of being helped somewhat. When we added controls for satisfaction to the models predicting perceived helpfulness (Table 4), all significant predictors became nonsignificant except for direct private insurance.

## Discussion

Across 5,248 patient-provider observations from 17 countries, we found high levels of patient-reported satisfaction and perceived helpfulness with mental health care received in the past year – 66–88% said they were at least satisfied with the treatment and services they received from providers and 64–90% said they were helped at least somewhat by providers. That said, considerably fewer said they were very satisfied (19-57%) or helped a lot (32-68%). The high levels of satisfaction and helpfulness observed in this study are consistent with previous research [12, 15, 38–41]. The relatively smaller group who endorsed ratings of very satisfied and helped a lot indicates that there is room for improvement, as do the small but important proportions who said they were very dissatisfied or not helped at all.

A goal of this study was to elucidate predictors of different levels of satisfaction and helpfulness in the context of mental health services. We found that the predictors of satisfaction and helpfulness were for the most part the same. Type of provider seen and type of mental disorder were important predictors, but the exact nature of associations differed depending on the type and level of outcome. With respect to providers seen, we found significant variation in levels of patient reported satisfaction and helpfulness across different types of providers. Notably, in our base regression models controlling for socio-demographic, clinical and treatment factors, spiritual advisors and to a lesser extent healers generally had the highest ratings, whereas social workers seen in a human services setting had the lowest ratings. The associations with being very satisfied persisted after controlling for helpfulness, but the associations with perceived helpfulness became nonsignificant when controlling for satisfaction.

One possible explanation for the high satisfaction with spiritual advisors and healers is that these types of providers offer kinds of support and opportunities for interaction beyond those usually available in formal healthcare [42] and/or provide an avenue for care to some people with few or no alternatives [43]. Another possibility is that the ethnic/cultural match of patients with spiritual advisors and healers is greater than the match for healthcare professionals. Connections with spiritual advisors and healers may be more strongly determined by initial beliefs and faiths associated with specific advisors. Satisfaction and helpfulness might not apply to individuals without those beliefs and faiths. In short, there might be quite different determinants in the paths leading to professional mental health care professionals and spiritual advisors and healers. Because advisors and healers are part of the services that are provided, more work is warranted to understand the processes and outcomes of these providers.

One possible explanation for the low satisfaction with social workers in human services settings is that patients seen in such settings are involved in types of issues, such as those involving welfare and possibly involuntary participation related to child protection or offending, that account for the low satisfaction. The WMH survey questions did not enquire about these possibilities, making it important to be cautious in interpreting this result as indicating that social workers in human services are less able than other providers in other settings to provide effective mental healthcare treatment. This should be part of a more general recognition that people with different types of characteristics not measured here vary in the types of providers from whom they seek help and the settings of those treatments, making it hazardous to interpret our results as providing clear evidence about comparative effectiveness of providers and/or settings.

We also found consistently that the lowest levels of satisfaction and perceived helpfulness among provider types were with social workers in a human services setting. Satisfaction and perceived helpfulness were also low for general medical doctors, whereas satisfaction but not perceived helpfulness was low for psychiatrists. In addition, satisfaction controlling for helpfulness was significantly lower for both general medical doctors and psychiatrists. These patterns suggest that the low satisfaction with these two types of providers might be due to higher expectations for being helped than for other types of providers.

Although previous research suggests that older patients are generally less satisfied than others [22, 33, 41, 44–48], we failed to find such an association. On the other hand, we found consistently that patients with a high level of education were more likely to be satisfied and to perceive themselves as having been helped than those with lower levels of education. The data available in the WMH surveys do not allow us to shed any light on the extent to which these patterns might be due to actual differences in the care received, patients’ expectations or attitudes about care, or other factors. However, we found that these associations diminished after adjusting for the proximal effects of helpfulness on satisfaction and vice versa. This suggests that high education might predispose patients towards a more positive perspective of care generally. Notwithstanding the cross-sectional nature of this study, in practical terms, this may suggest that strategies to improve satisfaction among less well-educated patients could also improve patient-defined outcomes for those same groups.

With respect to disorders, we found stronger associations involving perceived helpfulness than satisfaction. Patients with major depressive disorder and bipolar spectrum disorder were significantly less likely than others to report both being very satisfied (although aggregate variation across all disorders was not statistically significant) and being helped a lot. In addition, patients with bipolar spectrum disorder were significantly less likely than others with the same level of satisfaction to report being helped a lot. The same pattern held for social phobia. The number of comorbid mental disorders, in comparison, was not associated significantly with either satisfaction or perceived helpfulness. Although associations of disorder types with the outcomes were not due to differences in treatment providers, as the latter were controlled, it might be that comparative satisfaction and/or perceived helpfulness by disorder type varied across sectors. The latter possibility could not be examined here, though, as the sample was not large enough to support the estimation of interactions between disorders and provider types. Our study focused on services for mental disorders. It would be useful to understand if these findings characterized services for physical disorders. Although we are unaware of any such research, findings across types of disorders might extend the ability to match interventions and services with patient view of their care.

Research on personalized (precision) treatment currently emphasizes matching treatment techniques to characteristics of patients and providers. Our findings raise the prospective of whether satisfaction and helpfulness may play a role in that matching process. A methodological and clinical challenge may be to integrate factors that predict satisfaction and helpfulness into decisions about what treatments to provide to whom, in what settings, and by what professional or nonprofessional provider. However, attempting to propose such interpretation goes beyond our data because we did not evaluate treatment techniques. Moreover, the techniques that were used were likely to vary with (be confounded by) provider. Even so, both satisfaction and helpfulness warrant further attention to evaluate their determinants and their roles in both treatment participation, treatment outcomes, and the implications of these patterns for treatment matching.

### Limitations

To our knowledge, no other study has taken a dimensional approach to exploring the relationship between satisfaction and perceived helpfulness of mental healthcare in the way we did here. Other strengths of this study were its broad geographic coverage, including countries where patient perspectives are less commonly studied. However, there were limitations. Patient ratings of satisfaction and perceived helpfulness were based on single questions; we do not know how patients determined whether they were satisfied or helped by a given provider. The WMH surveys are cross-sectional, therefore we cannot establish causal pathways for the associations we observed. We focused on care received from providers; we could not include other services (such as self-help groups, internet self-help applications and hotlines) because the questions about satisfaction and helpfulness of these services were not asked in the surveys. There may have been unmeasured variables that played a part in determining satisfaction and helpfulness. For example, patients who have higher expectations of care might have lower levels of satisfaction than otherwise similar patients. Assessment of cross-national differences was not attempted given the complexities of assuming constancy of meaning of questions about satisfaction and helpfulness in different languages.

## Conclusions

This study addressed a gap in knowledge about the relationship between patient reported assessments of satisfaction and helpfulness of mental health care. The strong and positive association between satisfaction and helpfulness and their overlapping but not identical predictors suggests that these constructs are partly related, at least when measured with single global measures. Findings specifically suggest that factors such as education may predispose patients towards a more positive or negative perspective of care generally. In contrast, factors such as the type of provider seen might more strongly influence how satisfied patients are with treatment, while indicators of mental health status might more strongly influence how helpful they perceive treatment to be. A significant but understudied facet of service delivery is the role of spiritual advisors and healers. More research is needed to understand the similarities and differences in the paths toward different service elements and their outcomes. More generally, satisfaction and helpfulness remain key domains to be integrated in treatment evaluations because of their broader implications for attending and remaining in treatment and seeking additional treatment if an initial treatment is unsatisfactory or unhelpful.

### Electronic supplementary material

Below is the link to the electronic supplementary material.

Supplementary Material 1

## Data Availability

Access to the cross-national World Mental Health (WMH) data is governed by the organizations funding and responsible for survey data collection in each country. These organizations made data available to the WMH consortium through restricted data sharing agreements that do not allow us to release the data to third parties. The exception is that the U.S. data are available for secondary analysis via the Inter-University Consortium for Political and Social Research (ICPSR), http://www.icpsr.umich.edu/icpsrweb/ICPSR/series/00527.

## Abbreviations

WMH: World Mental Health
CIDI: Composite International Diagnostic Interview Version
DSM: IV–Diagnostic and Statistical Manual of Mental Disorders, Fourth Edition
OLS: Ordinary least squares
SAS: Statistical Analysis System
12M: 12–month
