# Supplementary material for: Factors associated with satisfaction and perceived helpfulness of mental healthcare: a World Mental Health Surveys report
Source: Int J Ment Health Syst. 2024 Mar 1;18:11. doi: 10.1186/s13033-024-00629-7 (PMC10908125; doi:10.1186/s13033-024-00629-7)
Supplement: Supplementary file 1 — Supplementary Material 1 [file 13033_2024_629_MOESM1_ESM.docx]

**Additional File 1**

*Factors associated with satisfaction and perceived helpfulness of mental healthcare: A World Mental Health Surveys report*

| **Supplementary Table 1. World Mental Health sample characteristics by World Bank income categories^a^** | | | | | | | | | | |
| --- | --- | --- | --- | --- | --- | --- | --- | --- | --- | --- |
|  | | | | | | | | | | |
| **Country by income category** | **Survey^b^** | **Sample characteristics^c^** | **Field Dates** | **Age range** | **Sample Size Part II** | **Response rate (%)^d^** | **Any DSM-IV 12-month disorder among the Part II sample** | | **12-month use of providers for mental health among those with any DSM-IV 12-month disorder** | |
|  |  |  |  |  |  |  | **%** | **(SE)** | **%** | **(SE)** |
| **I. Low/middle-income countries** | | | | | | | | | | |
| Brazil - São Paulo | São Paulo Megacity | São Paulo metropolitan area. | 2005-8 | 18-93 | 2,942 | 81.3 | 21.5 | (0.7) | 23.2 | (1.2) |
| Bulgaria 2 | NSHS - 2 | Nationally representative. | 2016-17 | 18-91 | 578 | 61.0 | 6.1 | (1.4) | 12.1 | (4.3) |
| Colombia – Medellin | MMHHS | Medellin metropolitan area | 2011-12 | 19-65 | 1,673 | 97.2 | 15.2 | (1.2) | 15.8 | (2.0) |
| Iraq | IMHS | Nationally representative. | 2006-7 | 18-96 | 4,332 | 95.2 | 8.1 | (0.6) | 10.5 | (2.7) |
| Mexico | M-NCS | All urban areas of the country (approximately 75% of the total national population). | 2001-2 | 18-65 | 2,362 | 76.6 | 11.0 | (0.8) | 17.3 | (1.9) |
| Peru | EMSMP | Five urban areas of the country (approximately 38% of the total national population). | 2004-5 | 18-65 | 1,801 | 90.2 | 9.5 | (0.6) | 18.2 | (2.2) |
| PRC^e^ – Shenzhen^f^ | Shenzhen | Shenzhen metropolitan area. Included temporary residents as well as household residents. | 2005-7 | 18-88 | 2,475 | 80.0 | 3.8 | (0.5) | 6.9 | (2.0) |
| Romania | RMHS | Nationally representative. | 2005-6 | 18-96 | 2,357 | 70.9 | 5.7 | (0.5) | 20.0 | (2.6) |
| **TOTAL** |  |  |  |  | (18,520) | 81.9 | 10.4 | (0.3) | 17.4 | (0.8) |
| **II. High-income countries** | | | | | | | | | | |
| Argentina | AMHES | Eight largest urban areas of the country (approximately 50% of the total national population) | 2015 | 18-98 | 2,116 | 77.3 | 11.5 | (0.7) | 26.9 | (2.9) |
| Japan | WMHJ 2002-2006 | Eleven metropolitan areas. | 2002-6 | 20-98 | 1,682 | 55.1 | 6.1 | (0.6) | 26.2 | (2.5) |
| New Zealand^f^ | NZMHS | Nationally representative. | 2004-5 | 18-98 | 7,312 | 73.3 | 18.8 | (0.5) | 35.7 | (1.3) |
| N. Ireland | NISHS | Nationally representative. | 2005-8 | 18-97 | 1,986 | 68.4 | 22.5 | (1.3) | 50.6 | (2.6) |
| Poland | EZOP | Nationally representative | 2010-11 | 18-65 | 4,000 | 50.4 | 8.5 | (0.4) | 18.2 | (1.7) |
| Portugal | NMHS | Nationally representative. | 2008-9 | 18-81 | 2,060 | 57.3 | 19.3 | (1.0) | 36.9 | (2.2) |
| Saudi Arabia^f^ | SNMHS | Nationally representative | 2013-16 | 18-65 | 1,793 | 61.0 | 11.4 | (1.1) | 17.2 | (2.9) |
| Spain-Murcia | PEGASUS- Murcia | Murcia region. Regionally representative. | 2010-12 | 18-96 | 1,459 | 67.4 | 12.7 | (0.9) | 40.9 | (3.0) |
| United States | NCS-R | Nationally representative. | 2001-3 | 18-99 | 5,692 | 70.9 | 22.3 | (0.8) | 39.3 | (1.2) |
| **TOTAL** |  |  |  |  | (28,100) | 63.5 | 14.7 | (0.3) | 35.7 | (0.8) |
| **III. POOLED ACROSS ALL COUNTRIES** | |  |  |  | (46,620) | 69.4 | 13.9 | (0.2) | 30.3 | (0.6) |
| Between countries, *X*^2^_16_ (p-value) | | |  |  |  |  | 1542.34 (< 0.001)* | | 399.15 (<0.001) * | |
| Low/middle-income countries vs. high-income countries, *X*^2^_1_ (p-value) | | |  |  |  |  | 1081.29 (< 0.001)* | | 217.17 (<0.001) * | |
|  | | | | | | | | | | |

* Significant at .05 level, two-sided test.

^a^ The World Bank (2012) Data. Accessed May 12, 2012 at: https://data.worldbank.org/country. Some of the WMH countries have moved into new income categories since the surveys were conducted. The income groupings above reflect the status of each country at the time of data collection. The current income category of each country is available at the preceding URL.

^b^ NSHS (Bulgaria National Survey of Health and Stress); MMHHS (Medellín Mental Health Household Study); IMHS (Iraq Mental Health Survey); M-NCS (The Mexico National Comorbidity Survey); EMSMP (La Encuesta Mundial de Salud Mental en el Peru); RMHS (Romania Mental Health Survey); AMHES (Argentina Mental Health Epidemiologic Survey); WMHJ 2002-2006 (World Mental Health Japan Survey); NZMHS (New Zealand Mental Health Survey); NISHS (Northern Ireland Study of Health and Stress); EZOP (Epidemiology of Mental Disorders and Access to Care Survey); NMHS (Portugal National Mental Health Survey); SNMHS (Saudi National Mental Health Survey); PEGASUS-Murcia (Psychiatric Enquiry to General Population in Southeast Spain-Murcia); NCS-R (The US National Comorbidity Survey Replication).

^c^ Most WMH surveys are based on stratified multistage clustered area probability household samples in which samples of areas equivalent to counties or municipalities in the US were selected in the first stage followed by one or more subsequent stages of geographic sampling (e.g., towns within counties, blocks within towns, households within blocks) to arrive at a sample of households, in each of which a listing of household members was created and one or two people were selected from this listing to be interviewed. No substitution was allowed when the originally sampled household resident could not be interviewed. These household samples were selected from Census area data. In Poland and Spain-Murcia, respondents were selected from municipal, country resident or universal health-care registries, without listing households. The Japanese sample is the only totally un-clustered sample, with households randomly selected in each of the 11 metropolitan areas and one random respondent selected in each sample household. 9 of the 17 surveys are based on nationally representative household samples.

^d^ The response rate is calculated as the ratio of the number of households in which an interview was completed to the number of households originally sampled, excluding from the denominator households known not to be eligible either because of being vacant at the time of initial contact or because the residents were unable to speak the designated languages of the survey. The weighted average response rate is 69.4%.

^e^ For the purposes of cross-national comparisons we limit the sample to those 18+.
